# Editorial: Optimizing GLP-1 receptor agonist use: mechanisms, clinical applications, and safety profiles

**DOI:** 10.3389/fphar.2026.1887751

**Published:** 2026-06-12

**Authors:** Karuna Rasineni, Patricia Sue Grigson

**Affiliations:** 1 Research Service, Veterans Affairs Nebraska-Western Iowa Healthcare System, Omaha, NE, United States; 2 Department of Biochemistry and Molecular Biology, University of Nebraska Medical Center, Omaha, NE, United States; 3 Penn State College of Medicine, Pennsylvania State University, Hershey, PA, United States

**Keywords:** alcohol use disorder (AUD), diabetes, GLP-1 receptor agonists, glucagon like peptide 1 (GLP-1), inflammation, obesity, steatotic liver disease (SLD)

## Introduction

Metabolic diseases, such as obesity and Type 2 diabetes mellitus (T2DM), represent a staggering global burden of morbidity and mortality. This risk is further compounded when these conditions are combined with alcohol or other disease states, which elevate mortality through multiple interconnected biological pathways. While core preventive strategies for metabolic disease involve caloric restriction, exercise, and abstinence, effective treatment requires agents that can restore metabolic homeostasis by acting at both the central and peripheral levels.

Amidst this urgent crisis, new hope has emerged with the development of long-acting glucagon-like peptide-1 receptor agonists (GLP-1RAs), a transformative class of drugs for these often-comorbid conditions. The papers compiled in this issue offer an exploration of the multifaceted role of GLP-1RAs for the treatment of not only T2DM and obesity, but also for the treatment of other disease states, from the periphery to the brain.

Specifically, the papers in this issue delve into the safety, efficacy, and cost of various GLP-1RAs, including novel formulations. Papers in the issue broaden the discussion of metabolic health by examining the effect of alcohol on plasma GLP-1 levels following metabolic surgery and by examining the protective effects of GLP-1RA treatment on vital organ systems, including the reward centers in the brain, the eye, the cardiovascular system, liver diseases, and mitigating breast cancer-related lymphedema. Finally, through a meta-analysis and data from the FDA Adverse Events Reporting System (FAERS), additional papers in this issue provide essential insight into the incidence of gastrointestinal and other adverse events associated with GLP-1RA use. Collectively, this research highlights the significant anti-inflammatory, antioxidant, and neuroprotective properties of these agents, showing GLP-1RAs to be far more than just glucose-lowering drugs.

## Deciphering mechanisms: central and peripheral action

In this collection, the paper by Li et al. reviews the structural and functional characteristics of the GLP-1R and how it works for the treatment of obesity, T2DM, and other metabolic disorders. In particular, the review outlines the timeline of discovery and clinical use of GLP-1RAs, describes how GLP-1 binds to GLP-1Rs in pancreatic cells to increase insulin synthesis and secretion and to inhibit glucagon release, describes underlying signaling pathways, and lists major clinical trials testing GLP-1RAs for the treatment of T2DM and obesity.

At the central level, the manuscript by Edvardsson et al. investigates the neurobiological effects of GLP-1RAs because, along with known peripheral effects, GLP-1 is also produced in single cells in the nucleus of the solitary track (NTS) and these cells project widely throughout the brain. The present study showed that the GLP-1RA, Ex-4, when administered systemically, increased metabolites of dopamine (DA) and serotonin (5HT) in the nucleus accumbens (NAc) shell, without affecting levels of DA, 5HT, norepinephrine (NE) or glutamate. Levels of serine, glycine, and taurine, on the other hand, were reduced. Some effects of systemically administered Ex-4 were mirrored by direct administration of Ex-4 into the NTS, including the increase in the serotonin metabolite, 5HIAA, and the decrease in both glycine and taurine. Given these findings, the authors conclude that some of the effects of systemic Ex-4 on the NAc shell are mediated by GLP-1Rs in the NTS, while other effects of Ex-4 on the NAc shell are mediated by action on GLP-1Rs elsewhere in the brain.

## Prescribing dynamics and new formulations

With a growing number of GLP-1RA formulations available, differences in cost, efficacy, and adverse event profiles can make it challenging for physicians to determine which GLP-1RA is best for a given patient. Addressing this, Li et al. describe the use of the mini-Health Technology Assessment (mini-HTA) to compare four once-weekly GLP-1R agonists prescribed in China for the treatment of T2DM (semaglutide, dulaglutide, exenatide microspheres, and polyethylene glycol [PEG] loxenatide), ranking them primarily on pharmaceutical properties, efficacy, safety, and cost in an effort to help guide prescribing practices for physicians.

Further, although there is an increasing number of GLP-1RAs approved for treatment, the trek toward development of novel GLP-1RAs with improved efficacy and safety profiles continues. To this end, Lin et al. demonstrate in a phase I clinical trial that beinaglutide, injected three times daily 5 min before mealtime, exhibited the expected safety profile with mild nausea and dizziness, a linear pharmacokinetic (PK) profile across increasing doses, and no accumulation in plasma levels over time. These phase I clinical trial data suggest that beinaglutide, the only GLP-1R agonist identical to human GLP-1, and already approved for the treatment of T2DM, has a safety profile that also is favorable for the treatment of overweight and obesity.

## Alcohol, surgery, and endocrine responses

In this issue, Molina-Castro et al. show that alcohol intake induces more rapid and higher peak blood alcohol levels following metabolic surgery (Roux-en-Y-gastric bypass [RYGB]) and sleeve gastrectomy [SC]). Critically, the inhibitory effect on plasma GLP-1, and the facilitative effect on plasma FGF21 (a liver-derived hormone implicated in glucose regulation and alcohol consumption) were sustained post-surgery despite dramatic bodyweight loss. These data are important given that both RYGB and SG can reportedly lead to an increase in alcohol use disorder (AUD) https://pubmed.ncbi.nlm.nih.gov/40925519/, although there is some evidence that risk of AUD may be less following SG than RYGB https://pubmed.ncbi.nlm.nih.gov/28802901/.

## Beyond metabolism: protective organ effects

Crucially, several studies evaluate the wide-ranging protective effects of GLP-1RAs on vital organ systems. Within this context, Mahalingam et al. provide a timely and comprehensive review that predicts the expansion of therapeutic utility beyond metabolic dysfunction alone. They propose GLP-1RAs as multifunctional therapeutics for the broader spectrum of steatotic liver disease (SLD), suggesting their use for conditions beyond metabolic dysfunction-associated steatotic liver disease (MASLD). Their work highlights the potential of these agents to mitigate liver injury in alcohol-associated liver disease (ALD), the hybrid condition metabolic dysfunction and alcohol-associated liver disease (MetALD), and even viral hepatic diseases. By exploring the mechanisms by which GLP-1RAs reduce hepatic steatosis, inflammation, and fibrosis, this review underscores the drugs' potential to disrupt progression toward hepatocellular carcinoma (HCC), regardless of whether the underlying insult is metabolic, alcoholic, or viral in origin.

For ocular therapeutics, the manuscript by Xie et al. describes how GLP-1RAs provide anti-inflammatory, antioxidant, and neuroprotective effects on the retina and other organs. These actions are achieved through suppression of proinflammatory cytokines, a reduction in mitochondrial dysfunction, a reduction in reactive oxygen species, inhibition of neurotoxic astrocyte activation, and restoration of inhibitory balance by increasing GABA release. The data presented in this manuscript are promising for the use of GLP-1RAs for the treatment of conditions beyond T2DM and obesity including glaucoma, diabetic retinopathy, and others, such as Alzheimer’s disease, Parkinson’s disease, and osteoarthritis, for example.

Regarding the cardiovascular system**,** Wu et al. provided a systematic review and meta-analysis by using data from 30 randomized clinical trials (RCTs) and 32,490 patients to examine the effect of treatment with the GLP-1RA, semaglutide, on cardiovascular health. Their results showed a reduction in the incidence of atrial fibrillation, complete atrioventricular block, death from cardiovascular causes, and revascularization in people treated with semaglutide for their T2DM. Importantly, improvement was not seen with all cardiovascular outcomes, and some positive outcomes were dependent upon the route of administration (oral or subcutaneous).

Strikingly, two papers address the use of GLP-1RAs for breast cancer-related lymphedema, a condition currently lacking a cure. A case report presented by Crowley et al. describes a 44-year-old female whose semaglutide treatment led to a 24% loss of bodyweight and a commensurate improvement in her lymphedema, allowing her to stop daily compression. This finding was substantiated by another study by Brown et al. using data from 3,830 cancer patients who underwent axillary lymph node dissection at Memorial Sloan Kettering Cancer Center. Results showed that treatment with a GLP-1RA led to an 86% reduction in the likelihood of developing lymphedema in patients with T2DM compared to a non GLP-1RA treated control group. A similar finding was obtained following the same surgery in non-diabetic patients treated with a GLP-1RA.

## Adverse events and risk stratification

These promising drugs, however, are not without side effects. The manuscript from Xie et al. presented a Bayesian network meta-analysis using the data from 48 random clinical trials to examine the effect of multiple GLP-1RAs on gastrointestinal (GI) adverse events (AEs), the most common side effects in patients with T2DM. Results show that tirzepatide ranked first for nausea, diarrhea, indigestion, and appetite loss. Semaglutide ranked first for constipation and second for nausea. Lixisenatide ranked first for vomiting. Loxenatide ranked second for diarrhea and vomiting, but did not rank at all for indigestion, constipation or appetite loss. Exenatide ranked sixth for most GI AEs, and Dulaglutide ranked seventh for nausea and vomiting. He et al. added to these findings by using data from the FDA Adverse Events Reporting System (FAERS) database and found that dehydration was the most frequent AE contributing to serious outcomes for several GLP-1RAs, alongside varied reports of decreased and increased appetite, food cravings, hypoglycemia, and food aversions. These AE profiles, then, can be used to best match a particular GLP-1RA with a given patient.

Collectively, the research compiled in this issue highlights the significant anti-inflammatory, antioxidant, and neuroprotective properties of these agents, painting a comprehensive picture of GLP-1RAs as far more than just glucose-lowering drugs. GLP-1RAs were found to improve disease states involving the liver, the eye, the cardiovascular system, and cancer-related lymphedema. Potential adverse effects, however, were also noted, reinforcing the recommendation that treatment with a GLP-1RA, for any reason, should be monitored by a physician.

